# Age-Dependent Effects of Graphene Oxide on Hydration-Gel Evolution and Strength Development of Fly Ash-Blended Cementitious Materials

**DOI:** 10.3390/gels12040312

**Published:** 2026-04-06

**Authors:** Hai-Sheng Huang, Ying Peng, Xiu-Cheng Zhang, Xue-Fei Chen

**Affiliations:** 1 School of Civil Engineering, Putian University, Putian 351100, China; 2 Engineering Research Center of Disaster Prevention and Mitigation of Southeast Coastal Engineering Structures (JDGC03), Fujian Province University, Putian 351100, China; 3 Department of Architecture and Civil Engineering, City University of Hong Kong, 83 Tat Chee Avenue, Kowloon Tong 999077, Hong Kong, China

**Keywords:** waste, gels, cementitious materials

## Abstract

Fly ash is an effective supplementary cementitious material for reducing clinker consumption and carbon emissions, but its low early reactivity often results in delayed hydration and insufficient early-age strength. This study investigated the age-dependent role of graphene oxide (GO) in fly ash-blended cementitious materials by combining compressive strength testing with X-ray diffraction (XRD), thermogravimetric analysis (TG-DTG), ^29^Si magic-angle spinning nuclear magnetic resonance (^29^Si MAS NMR), and scanning electron microscopy coupled with energy-dispersive spectroscopy (SEM-EDS). Fly ash replacement levels of 10%, 20%, and 30% were considered, and 0.07% GO was introduced to evaluate its effect at 3, 7, and 28 days. The results showed that fly ash reduced the 3-day compressive strength, whereas the strength differences became much smaller at 28 days. GO enhanced the compressive strength of all fly ash-blended mixtures. XRD and TG-DTG results showed that GO refined Ca(OH)_2_ crystallization and reduced the retained CH content, indicating more effective CH utilization during hydration and pozzolanic reaction. At 28 days, the incorporation of 0.07% GO increased the compressive strength of the 30% fly ash mixture from 47.38 MPa to 56.58 MPa, while reducing the total CH content from 14.20% to 12.89%, indicating enhanced CH utilization and gel development. ^29^Si MAS NMR further demonstrated that GO promoted a more mature and polymerized silicate gel structure, as evidenced by lower Q^0^ fractions, higher mean chain length, and higher proportions of more polymerized silicate species. SEM-EDS observations confirmed that GO led to a denser matrix, less dominant coarse CH, and lower Ca/Si and Ca/(Si + Al) ratios. Overall, GO improved the mechanical performance of fly ash-blended cementitious materials through coupled regulation of hydration products, silicate gel polymerization, and matrix densification.

## 1. Introduction

The cement industry is widely recognized as one of the major contributors to global anthropogenic carbon emissions, primarily due to the calcination of limestone and the energy-intensive production of Portland cement clinker. In this context, the partial replacement of cement with supplementary cementitious materials has become an effective and widely adopted strategy for reducing the environmental burden of cement-based materials while promoting the valorization of industrial by-products [1,2,3,4,5]. Among various supplementary cementitious materials, fly ash has attracted sustained attention because of its abundant availability, low cost, spherical particle morphology, and long-term pozzolanic reactivity. The incorporation of fly ash can not only reduce clinker consumption and associated CO_2_ emissions, but also improve the later-age performance, dimensional stability, and durability of cementitious systems by refining the hydration products and moderating the internal pore solution chemistry. Although fly ash may contain trace heavy metals originating from coal combustion, its use in cementitious materials is generally considered an effective valorization route because the cement matrix can substantially immobilize these species and reduce their environmental mobility. Its environmental benefit is thus as a conditional advantage derived from both industrial waste reutilization and partial cement replacement, rather than as an inherently risk-free material. However, despite these advantages, the use of fly ash, especially at relatively high replacement levels, is commonly accompanied by several intrinsic drawbacks [6,7,8,9]. Owing to its lower early reactivity compared with Portland cement, fly ash-blended systems often exhibit retarded hydration, insufficient early gel formation, delayed microstructural build-up, and consequently reduced early-age mechanical strength. This limitation significantly restricts the broader engineering application of high-volume fly ash cementitious materials, particularly in structural and prefabricated components where early-age strength development is essential. Therefore, improving the early-age performance of fly ash-blended cementitious materials while preserving their long-term sustainability advantages remains a critical scientific and technological challenge.

To overcome the early-age limitations of fly ash systems, nanomaterial-based modification has emerged as a promising route in recent years, among which graphene oxide (GO) is considered especially attractive. GO possesses an ultrahigh specific surface area and is enriched with oxygen-containing functional groups, such as hydroxyl, carboxyl, and epoxy groups, which endow it with high surface activity and strong physicochemical interaction potential in alkaline cementitious environments [10,11,12,13]. These structural characteristics enable GO to participate in the hydration process not as a conventional reactive binder, but as a multifunctional nanoscale regulator. On the one hand, GO sheets may act as heterogeneous nucleation substrates for hydration products, thereby accelerating the deposition of calcium silicate hydrate and related gel phases during the early stages of hydration. On the other hand, the two-dimensional morphology and surface functionality of GO may influence the spatial distribution of hydration products, modify gel growth pathways, and promote the formation of a more continuous and compact microstructure. A number of previous studies have reported that GO can improve the compressive strength [14], flexural strength [15], toughness [16], and crack resistance [17] of cement-based materials, while in some cases also affecting rheological properties and setting behavior. These beneficial effects have generally been attributed to one or more mechanisms, including nucleation promotion, crack-bridging, micro-filling, and interfacial regulation. Nevertheless, the current understanding of GO in fly ash-blended systems is still far from complete. Many existing studies tend to focus on macroscopic performance enhancement, such as strength gain or workability variation, whereas the underlying physicochemical origin of these changes is often discussed only qualitatively. In particular, the role of GO in regulating hydration product evolution, CH consumption, secondary pozzolanic reaction, and silicate gel structural development in fly ash-containing systems has not yet been sufficiently clarified. Since the performance of blended cementitious materials is fundamentally governed by the evolution of their hydration products and gel structures across multiple scales, a deeper mechanistic investigation is required in order to move beyond phenomenological observations.

A further limitation in the existing literature lies in the insufficient integration of multiscale characterization techniques when evaluating the effect of GO on fly ash-blended cementitious materials. Although X-ray diffraction is commonly used to identify crystalline hydration products and thermogravimetric analysis is often employed to estimate bound water and calcium hydroxide contents, relatively few studies have systematically combined these methods with ^29^Si MAS NMR and SEM to elucidate the evolution of silicate gel structure and microstructural morphology in a coordinated manner. This is a crucial gap, because the principal hydration products responsible for strength development in blended cementitious systems, especially C-(A)-S-H type gels, are largely poorly crystalline or amorphous, and therefore cannot be fully interpreted by XRD alone [18,19,20]. Thermogravimetric analysis can provide complementary information on hydration progress and calcium hydroxide consumption [21,22,23], but it still cannot directly reveal how the local silicate environment evolves as hydration and pozzolanic reactions proceed. By contrast, ^29^Si MAS NMR is uniquely suited to characterizing the structural state of silicate species, including the distribution of Q^0^, Q^1^, Q^2^, and even higher-order silicate units, thereby offering direct insight into the degree of polymerization and chain development of hydration gels. When combined with SEM observations of morphology, matrix continuity, and the interaction between unreacted fly ash particles and surrounding gels, such a multi-technique framework can provide a much more convincing explanation for the strength evolution of GO-modified fly ash systems. In addition, another unresolved issue concerns whether the role of GO remains the same across different fly ash replacement levels. Because cement dilution, calcium availability, alkalinity, and pozzolanic potential all vary with fly ash dosage, GO is unlikely to exert an identical influence in systems with low and high fly ash contents. However, systematic comparative studies on this aspect remain limited. Another important gap lies in the insufficient understanding of the age dependence of GO action. Rather than treating the role of GO as two isolated mechanisms, it should be considered a time-dependent and continuous process. At early ages, GO mainly provides active sites for the precipitation of hydration products, thereby accelerating cement hydration and promoting initial strength development. As hydration proceeds, this early promotion effect gradually creates a more favorable microstructural environment for the subsequent pozzolanic reaction of fly ash. In this sense, the later-age contribution of GO is not an independent mechanism, but a continued manifestation of its early-age hydration promotion and microstructure refinement. Accordingly, the effect of GO at 3 days is primarily associated with nucleation promotion and accelerated initial gel formation, whereas at 28 days it is more closely reflected in intensified fly ash reaction, additional CH consumption, and further maturation of silicate gels. The transition between these stages should therefore be understood as a continuous evolution rather than an abrupt mechanistic shift. Nevertheless, such age-dependent coupling has rarely been discussed in depth. As a result, a coherent mechanistic framework linking hydration-product evolution, gel structural development, and strength development in GO-modified fly ash-blended cementitious materials is still lacking.

Therefore, this study aims to systematically investigate the age-dependent role of graphene oxide in fly ash-blended cementitious materials by focusing on three interrelated aspects. First, the evolution of hydration products is analyzed through XRD and TG in order to clarify how GO affects hydration progress and pozzolanic reaction at different ages and fly ash replacement levels. Second, the structural development of silicate gels is characterized by ^29^Si MAS NMR, together with SEM observations of morphological features and matrix densification, so as to reveal how GO regulates the formation, polymerization, and spatial organization of hydration gels. Third, the relationship between multiscale microstructural evolution and compressive strength is established to elucidate the mechanism by which GO compensates for the early-age weakness of fly ash-blended systems and contributes to their later-age performance. By integrating crystalline phase analysis, thermogravimetric evidence, molecular-scale silicate structural characterization, and microscale morphological observation, this work is expected to provide a more complete understanding of the physicochemical role of GO in fly ash cementitious matrices. The main contribution of this study is not simply to report strength improvement after GO incorporation, but to establish a multiscale interpretation linking compressive strength, CH evolution, silicate gel polymerization, and matrix densification in fly ash-blended cementitious materials. The findings may also offer theoretical support for the design of low-carbon, high-performance cementitious materials incorporating both nanomodification and industrial solid-waste utilization.

## 2. Results and Discussion

### 2.1. Compressive Strength

**Figure 1:**
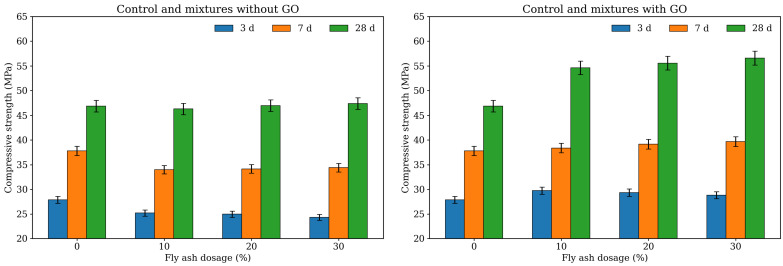
Compressive strength distributions of different mixtures at 3, 7, and 28 days.

Figure 1 presents the compressive strength results of all mixtures at 3, 7, and 28 days. In the present study, F denotes the fly ash replacement level of 10%, 20%, and 30%, while G7 represents the incorporation of 0.07% GO. Therefore, G0F10, G0F20, and G0F30 correspond to the mixtures without GO and containing 10%, 20%, and 30% fly ash, respectively, whereas G7F10, G7F20, and G7F30 denote the corresponding fly ash mixtures modified with 0.07% GO.

As shown in the figure, the compressive strength exhibited distinct dependences on curing age, fly ash dosage, and GO incorporation. For the reference mixture without fly ash and GO (G0), the compressive strength reached 27.90 MPa at 3 days, 37.79 MPa at 7 days, and 46.87 MPa at 28 days. After introducing fly ash without GO, the early-age strength decreased consistently. At 3 days, the strength declined from 27.90 MPa for G0 to 25.20, 24.96, and 24.33 MPa for G0F10, G0F20, and G0F30, respectively, indicating that increasing fly ash replacement led to a gradual reduction in early-age load-bearing capacity [24,25,26]. This reduction can be mainly attributed to the dilution effect of cement clinker and the relatively low early pozzolanic reactivity of fly ash, which resulted in slower hydration product accumulation and delayed structural build-up. A similar trend remained evident at 7 days, where the strengths of G0F10, G0F20, and G0F30 were 34.01, 34.16, and 34.41 MPa, respectively, all lower than that of G0. However, at 28 days, the differences between the fly ash mixtures without GO and the reference mixture became much smaller. The 28-day strengths of G0F10, G0F20, and G0F30 were 46.29, 46.98, and 47.38 MPa, respectively, which were very close to or slightly higher than that of G0. This result suggests that although fly ash hindered early-age strength development, its later pozzolanic contribution gradually compensated for the initial strength loss, leading to a recovery in compressive performance at 28 days.

The effect of GO on compressive strength was highly pronounced at all curing ages. For the mixtures containing GO, the 3-day compressive strengths of G7F10, G7F20, and G7F30 reached 29.75, 29.35, and 28.83 MPa, respectively. Compared with their GO-free counterparts, the strength increases were 18.06% for F10, 17.59% for F20, and 18.50% for F30. More importantly, all GO-modified fly ash mixtures exhibited even higher 3-day strength than the plain reference G0. This finding indicates that the incorporation of 0.07% GO not only compensated for the adverse effect of fly ash on early-age strength but also enabled the fly ash-blended systems to surpass the control without fly ash. Such behavior strongly suggests that GO played a crucial role in accelerating early hydration and promoting the initial formation of load-bearing gel phases.

At 7 days, the strengthening effect of GO became even more evident in absolute terms. The compressive strengths of G7F10, G7F20, and G7F30 were 38.38, 39.16, and 39.67 MPa, respectively, which exceeded those of G0F10, G0F20, and G0F30 by 12.85%, 14.64%, and 15.29%. In addition, all GO-containing mixtures also showed slightly higher values than G0 at the same age. This result implies that the beneficial role of GO was not limited to the very early stage but extended into the intermediate hydration period. From the rose diagram at 7 days, it can also be observed that the sectors corresponding to G7F10–G7F30 collectively expanded outward relative to both G0 and the GO-free fly ash mixtures, indicating a systematic and stable enhancement effect rather than an isolated improvement in one particular composition.

The most remarkable enhancement appeared at 28 days. The compressive strengths of G7F10, G7F20, and G7F30 increased to 54.60, 55.56, and 56.58 MPa, respectively, corresponding to increases of 17.95%, 18.25%, and 19.42% compared with G0F10, G0F20, and G0F30. Relative to the plain reference G0, the increases were also substantial, reaching 16.49%, 18.53%, and 20.71%, respectively. These results demonstrate that the effect of GO was sustained and even amplified with curing age. In particular, the gradual increase from G7F10 to G7F30 at 28 days suggests that, within the investigated fly ash range, the interaction between GO and fly ash became increasingly favorable at later age. This may indicate that GO not only accelerated the early hydration of cement particles, but also created a more suitable microchemical and structural environment for the subsequent pozzolanic reaction of fly ash, thereby leading to greater formation of secondary hydration products and a more mature gel network.

Another noteworthy feature is that the influence of fly ash dosage differed significantly depending on whether GO was present. In the absence of GO, increasing fly ash content from 10% to 30% caused a slight reduction in 3-day strength and only a marginal improvement at 28 days. This suggests that the positive later-age contribution of fly ash was relatively limited under the GO-free condition. In contrast, in the presence of GO, the strength values remained high across all fly ash levels and even exhibited a gradual increase with increasing fly ash content at 7 and 28 days. For example, at 28 days, the strength rose from 54.60 MPa for G7F10 to 56.58 MPa for G7F30. This trend indicates that GO enhanced the utilization efficiency of fly ash in the blended system. In other words, the role of fly ash was no longer dominated solely by clinker dilution but was increasingly associated with its later pozzolanic reactivity when GO was introduced.

From a mechanistic perspective, the strength enhancement induced by GO can be preliminarily attributed to several coupled effects. First, the oxygen-containing functional groups and ultrathin nanosheet structure of GO can provide abundant heterogeneous nucleation sites for hydration products, thereby accelerating the precipitation of early C–S–H and related gel phases [27,28]. Second, GO may promote a more uniform distribution of hydration products and improve the continuity of the solid skeleton, which is particularly beneficial for fly ash-blended systems that otherwise suffer from slow early microstructural development. Third, as curing proceeds, the accelerated early hydration and improved local reaction environment may facilitate the subsequent pozzolanic reaction of fly ash, leading to enhanced calcium hydroxide consumption and the formation of additional C-(A)-S-H gel. As a result, the later-age strength benefit becomes more pronounced than the early-age one. However, compressive strength alone cannot fully reveal the underlying physicochemical origin of these improvements. Therefore, the hydration product evolution and silicate gel structural development were further examined by XRD, TG, and ^29Si MAS NMR in the following sections.

### 2.2. Hydration Product Evolution Revealed by XRD

**Figure 2:**
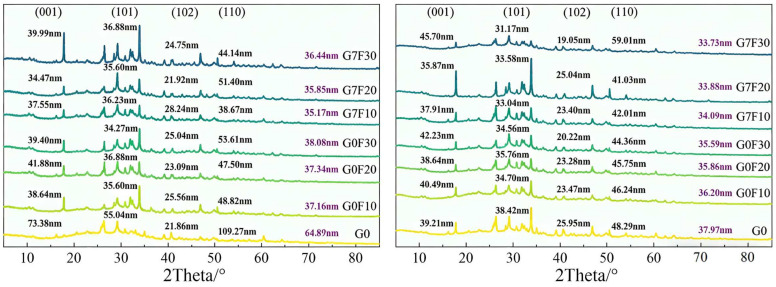
XRD patterns of fly ash blended samples and samples co-blended with GO at 3d age (**left**) and 28 d (**right**).

Figure 2 shows the XRD patterns of the fly ash-blended mixtures with and without GO at 3 and 28 days, together with the calculated crystallite sizes of Ca(OH)_2_ along different crystal planes. In general, all mixtures exhibited the characteristic diffraction features of hydrated cementitious systems, while the reflections associated with CH remained clearly identifiable. The XRD results indicate that both fly ash incorporation and GO addition significantly influenced the crystallization behavior of CH, and these changes became more evident when the average CH crystallite size was compared among the mixtures.

At 3 days, the plain reference mixture G0 exhibited the largest average CH crystallite size, reaching 64.89 nm. After the incorporation of fly ash, the average CH crystallite size decreased sharply to 37.16 nm for G0F10, 37.34 nm for G0F20, and 38.08 nm for G0F30. This substantial reduction suggests that the introduction of fly ash markedly suppressed the growth of coarse CH crystals at early age. Such a phenomenon can be attributed to two combined factors. First, the replacement of cement by fly ash reduced the effective clinker content, thereby lowering the amount of directly generated CH. Second, the presence of fly ash particles altered the nucleation and spatial distribution of hydration products, which hindered the development of large CH crystals and led to a more dispersed crystallization state [29,30,31]. Although the average CH crystallite sizes of the three fly ash mixtures were close to each other at 3 days, all of them remained far below that of G0, indicating that the influence of fly ash on CH crystallization was already pronounced even at the early stage.

**Figure 3:**
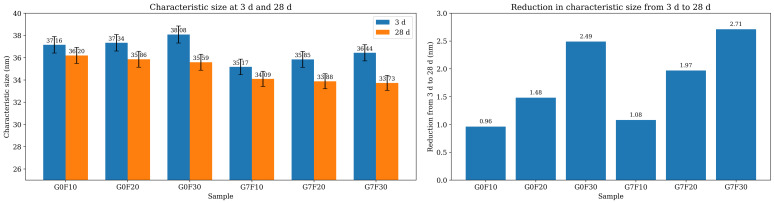
Characteristic size of CH (**left**) and the size reduction from 3 d to 28 d (**right**).

When GO was introduced into the fly ash-blended systems, the CH crystallite size decreased further (see Figure 3). At 3 days, the average values were 35.17 nm for G7F10, 35.85 nm for G7F20, and 36.44 nm for G7F30, all of which were lower than those of the corresponding GO-free mixtures. This result indicates that GO further inhibited the coarsening of CH crystals and promoted the formation of finer CH domains. The reduction in CH crystallite size in the GO-modified mixtures suggests that GO altered the precipitation behavior of hydration products, likely by providing abundant heterogeneous nucleation sites through its oxygen-containing functional groups and high specific surface area. Instead of allowing CH to grow into relatively large crystals, the presence of GO favored a more dispersed and refined hydration environment. This effect is important because the formation of finer CH crystals is usually associated with a more homogeneous microstructure and may indirectly facilitate the precipitation and spatial continuity of gel phases.

At 28 days, the differences in average CH crystallite size among the mixtures became smaller than those at 3 days, but the overall trend remained consistent. The average CH crystallite size of G0 decreased markedly to 37.97 nm, indicating that the CH crystals in the reference system underwent substantial refinement with ongoing hydration. For the fly ash mixtures without GO, the average CH crystallite sizes were 36.20 nm for G0F10, 35.86 nm for G0F20, and 35.59 nm for G0F30, showing a slight downward trend with increasing fly ash dosage. This result suggests that, at later age, fly ash continued to regulate the crystallization state of CH, likely through its progressively developed pozzolanic reaction, which consumed CH and reduced the tendency for large CH crystals to persist in the matrix. In the GO-containing mixtures, the average CH crystallite sizes further decreased to 34.09, 33.88, and 33.73 nm for G7F10, G7F20, and G7F30, respectively. Therefore, even at 28 days, GO still maintained its effect on CH refinement, and this influence became slightly stronger as the fly ash dosage increased.

A more detailed comparison between 3 and 28 days reveals an interesting age-dependent feature. For G0, the average CH crystallite size decreased dramatically from 64.89 to 37.97 nm, whereas the fly ash-containing systems exhibited much smaller changes over the same period. This implies that in the plain cement system, CH initially formed in a relatively coarse and concentrated manner, while the incorporation of fly ash, with or without GO, made the CH phase much finer from the outset. In other words, the combined presence of fly ash and GO modified the early crystallization pathway of CH rather than merely affecting its later transformation. This is consistent with the strength results above, where GO effectively compensated for the early-age strength loss induced by fly ash. The refined CH crystallization behavior observed here suggests that GO promoted a more favorable early hydration environment and avoided the formation of excessively coarse CH crystals that are generally less beneficial to matrix integrity.

In addition to crystallite refinement, the XRD results also imply that the effect of GO was not limited to a simple physical dispersion mechanism. The systematic reduction in CH crystallite size across all fly ash replacement levels suggests that GO influenced the hydration product evolution in a persistent and composition-dependent manner. At early age, this effect was likely associated with accelerated nucleation and more uniform precipitation of hydration products [32,33,34]. At later age, it may also be related to enhanced interaction between CH and fly ash reaction sites, thereby favoring the continuous development of secondary C-(A)-S-H type gels [35,36,37,38]. Therefore, the XRD evidence supports the view that GO regulated not only the morphology of CH crystals, but also the overall hydration environment of fly ash-blended cementitious materials. However, XRD mainly reflects the crystallization state of relatively crystalline phases and cannot fully quantify the total amount of CH or the extent of its consumption. For this reason, TG/DTG analysis was further conducted to examine the thermal decomposition behavior and total CH content of the different mixtures.

### 2.3. Thermogravimetric Evidence of Hydration and Pozzolanic Reaction

**Figure 4:**
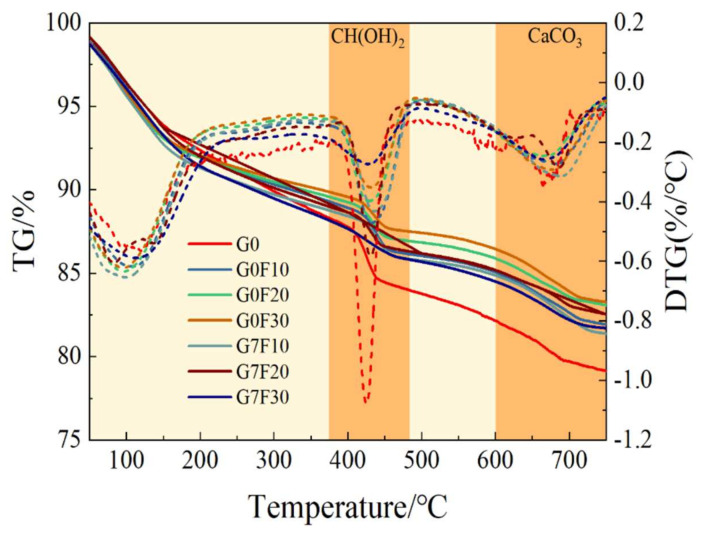
TG-DTG curves of fly ash-only samples and fly ash–GO co-doped samples.

**Figure 5:**
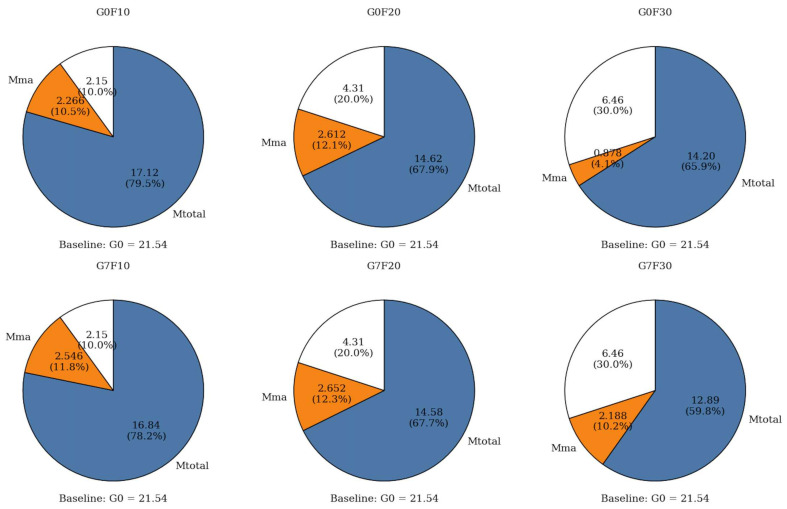
Partitioned pie-chart visualization of CH content in blended cementitious systems relative to the pure cement baseline. **Note:** the area in blue indicates the amount of remaining CH.

Figure 4 presents the TG-DTG curves of the fly ash-blended mixtures with and without GO at 28 days, and the corresponding total CH contents are presented in Figure 5. In the TG-DTG profiles, the main mass-loss regions can be identified as the dehydration of bound water and hydration products at low temperature, the decomposition of Ca(OH)_2_ in the range of approximately 400–450 °C, and the decomposition of CaCO_3_ at around 600–750 °C [39]. Among these regions, the mass loss associated with CH decomposition is particularly important because it directly reflects the amount of portlandite retained in the hydrated matrix and indirectly indicates the progress of hydration and pozzolanic reaction.

As illustrated by Figure 5, the total CH content of the plain reference sample G0 was 21.54%, which was the highest among all mixtures. After the incorporation of fly ash, the total CH content decreased significantly to 17.12% for G0F10, 14.62% for G0F20, and 14.20% for G0F30. This progressive reduction is consistent with the expected effect of fly ash in blended cementitious systems. On the one hand, the replacement of cement by fly ash reduced the clinker content and thus lowered the amount of CH directly produced by cement hydration. On the other hand, the pozzolanic reaction of fly ash gradually consumed CH to form additional secondary hydration products. As a result, increasing fly ash dosage led to a systematic decline in the retained CH content. This trend agrees well with the XRD observations, where the crystallization of CH became notably refined after fly ash incorporation.

The effect of GO on total CH content showed a dosage-dependent trend across the fly ash-blended systems. Compared with the corresponding GO-free mixtures, the total CH content slightly decreased from 17.12% to 16.84% for F10, from 14.62% to 14.58% for F20, and more noticeably from 14.20% to 12.89% for F30 after GO addition. Although the reductions for F10 and F20 were relatively small, the overall trend indicates that GO tended to lower the residual CH content, especially at the higher fly ash dosage. This result suggests that GO not only affected the crystallization state of CH, as demonstrated by XRD, but also influenced its net retention in the hydrated system. Since GO itself does not directly contribute CH, the lower CH content in the GO-modified mixtures is more reasonably interpreted as evidence of a more effective conversion of CH into secondary hydration products or a more efficient coupling between cement hydration and fly ash reaction.

To further distinguish the role of fly ash-related reaction in CH evolution, the calculated values of M_ce_ and M_MA_ were also compared. Here, M_ce_ represents the theoretical CH content generated solely by the cement fraction in the blended system, whereas M_MA_ reflects the CH variation associated with the mineral admixture. For the GO-free mixtures, M_MA_ values were 2.264%, 2.608%, and 0.882% for G0F10, G0F20, and G0F30, respectively. After GO incorporation, these values increased to 2.546%, 2.652%, and 2.186% for G7F10, G7F20, and G7F30, respectively. The increase in M_MA_, particularly for the 30% fly ash mixture, indicates that the CH associated with the fly ash-containing system was more strongly affected when GO was present. This implies that GO enhanced the interaction between fly ash and the surrounding hydration environment, making the fly ash-related reaction more pronounced. In other words, the role of GO appears to extend beyond accelerating cement hydration alone; it also promotes the effective participation of fly ash in the later-age reaction process.

The DTG curves provide further support for this interpretation. All mixtures showed a distinct endothermic decomposition feature in the CH region, but the depth and shape of the CH-related DTG trough varied among the samples. The reference mixture G0 displayed the most prominent CH decomposition signal, in agreement with its highest total CH content. By contrast, the fly ash-containing mixtures exhibited weakened CH-related DTG peaks, confirming that fly ash reduced the amount of retained portlandite. The GO-modified mixtures generally showed a further attenuation of the CH decomposition feature, especially at higher fly ash dosage, which is consistent with the reduced M_total_ values. Meanwhile, the decomposition region assigned to CaCO_3_ remained visible in all samples, indicating that partial carbonation occurred during sample preparation or storage, but this did not alter the overall conclusion regarding CH evolution.

When the TG/DTG results are considered together with the compressive strength data, an important feature becomes evident. The results show a clear quantitative trend between CH evolution and strength development, particularly at 28 days. For the GO-free fly ash mixtures, the total CH content decreased from 17.12% in G0F10 to 14.62% in G0F20 and 14.20% in G0F30, while the corresponding compressive strengths increased from 46.29 MPa to 46.98 MPa and 47.38 MPa. After GO incorporation, the same trend became more evident: the CH content decreased from 16.84% in G7F10 to 14.58% in G7F20 and 12.89% in G7F30, whereas the compressive strength increased from 54.60 MPa to 55.56 MPa and 56.58 MPa. In particular, for the 30% fly ash mixture, GO reduced the CH content from 14.20% to 12.89% while increasing the 28-day compressive strength from 47.38 MPa to 56.58 MPa, corresponding to a strength increase of 19.42%. This indicates that lower retained CH was associated with higher strength.

Overall, the TG/DTG analysis confirms three main points. First, increasing fly ash dosage reduced the total CH content because of both cement dilution and pozzolanic consumption. Second, GO further decreased the retained CH content, particularly in the high-fly-ash mixture, indicating a stronger coupling effect between GO and fly ash reaction. Third, when combined with the XRD evidence of reduced CH crystallite size, the TG results demonstrate that GO simultaneously refined the crystallization state of CH and promoted its further utilization in the blended system. These findings provide strong evidence that the strength enhancement induced by GO was closely related to its regulation of hydration product evolution and CH conversion behavior. To further clarify how these changes were reflected in the silicate environment of the binding gels, ^29^Si MAS NMR analysis is discussed in the following section.

### 2.4. Evolution of Silicate Gel Structure from ^29^Si MAS NMR

**Figure 6:**
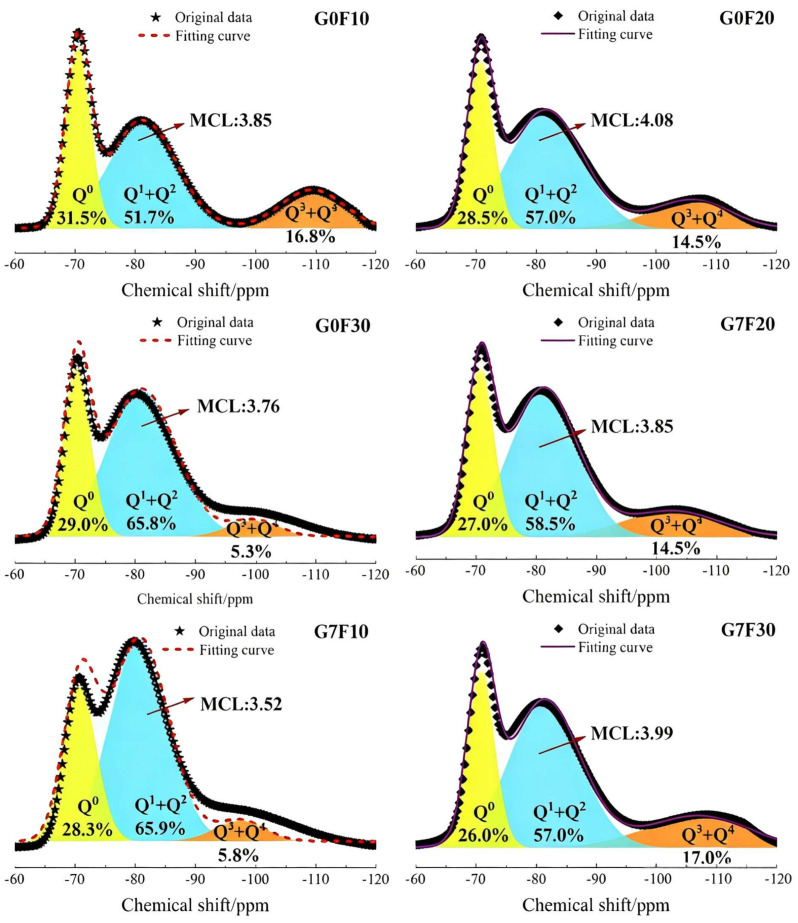
^29^Si NMR spectra of fly ash single-doped samples and samples co-doped with GO.

Figure 6 presents the ^29^Si MAS NMR spectra of the fly ash-blended mixtures with and without GO, and the corresponding deconvolution results reveal the relative fractions of Q^0^, Q^1^ + Q^2^, and Q^3^ + Q^4^ species together with the mean chain length (MCL). In general, the spectra of all fly ash-containing samples can be divided into three main regions: the resonance near −70 ppm assigned to Q^0^, which is mainly associated with unreacted or weakly reacted silicate units; the dominant band in the range of approximately −75 to −90 ppm corresponding to Q^1^ and Q^2^ species, which represent the chain-end and chain-middle silicate tetrahedra in C-(A)-S-H type gels; and the broader resonance at lower field attributed to Q^3^ and Q^4^, reflecting more polymerized and cross-linked silicate structures. Therefore, changes in the relative proportions of these structural units can directly reflect the evolution of gel polymerization and the degree of silicate chain development in the fly ash-blended cementitious system.

For the mixtures without GO, the fraction of Q^0^ decreased slightly with increasing fly ash dosage, from 31.5% for G0F10 to 29.0% for G0F20 and 28.3% for G0F30. Meanwhile, the proportion of Q^1^ + Q^2^ increased from 51.7% for G0F10 to 65.8% for G0F20 and 65.9% for G0F30. These results indicate that, as the fly ash content increased, the silicate environment became increasingly dominated by chain-like gel structures rather than isolated or poorly reacted silicate units. However, the fraction of Q^3^ + Q^4^ did not increase correspondingly; instead, it declined from 16.8% in G0F10 to 5.3% in G0F20 and 5.8% in G0F30. This suggests that although higher fly ash dosage promoted the formation of chain-like silicate units, the degree of highly polymerized or cross-linked silicate structures remained limited in the GO-free system. This interpretation is further supported by the MCL results, which decreased from 3.85 for G0F10 to 3.76 for G0F20 and 3.52 for G0F30. Therefore, in the absence of GO, increasing fly ash dosage did not continuously improve the overall polymerization level of the silicate network, but instead tended to shift the gel structure toward a Q^1^ + Q^2^-dominated state with a reduced mean chain length.

After GO incorporation, the silicate gel structure evolved in a clearly different manner. The Q^0^ fraction further decreased to 28.5%, 27.0%, and 26.0% for G7F10, G7F20, and G7F30, respectively, all lower than those of their GO-free counterparts. This reduction indicates that GO promoted the conversion of less-reacted silicate species into more integrated gel structures. More importantly, the MCL values increased to 4.08, 3.85, and 3.99 for G7F10, G7F20, and G7F30, respectively, compared with 3.85, 3.76, and 3.52 for G0F10, G0F20, and G0F30. The increase in MCL demonstrates that GO favored the development of longer silicate chains and a more mature gel structure. The enhancement was particularly pronounced in the 30% fly ash mixture, where the MCL increased from 3.52 to 3.99 after GO addition, indicating that GO effectively compensated for the structural limitation of the high-fly-ash system.

The variation in the Q^3^ + Q^4^ fraction provides additional evidence of this GO-induced effect. For G7F10 and G7F20, the Q^3^ + Q^4^ content was 14.5%, which, although slightly lower than that of G0F10, was markedly higher than those of G0F20 and G0F30. In G7F30, the Q^3^ + Q^4^ fraction further increased to 17.0%, which was substantially higher than the 5.8% observed in G0F30. This result strongly suggests that GO promoted the formation of more polymerized silicate environments, particularly in the high fly ash mixture. In other words, GO did not merely increase the amount of chain-like gel species but also facilitated the evolution of the gel network toward a more connected and polymerized state. Such a structural change is highly consistent with the substantial 28-day strength enhancement observed in Section 2.1, especially for G7F30.

Another notable feature is that the role of GO was not simply to increase the Q^1^ + Q^2^ fraction. In fact, compared with the GO-free mixtures, the Q^1^ + Q^2^ content in the GO-containing systems decreased from 65.8% to 58.5% for F20 and from 65.9% to 57.0% for F30. However, this decrease was accompanied by a simultaneous increase in Q^3^ + Q^4^ and MCL. This indicates that GO promoted the transformation of part of the chain-like Q^1^ + Q^2^ units into more polymerized silicate structures, rather than merely increasing the quantity of intermediate gel species. Therefore, the main contribution of GO to gel structural development lies in improving the maturity and polymerization degree of the silicate network, not only in accelerating its initial formation. This point is important because it helps explain why GO-modified mixtures exhibited not only improved early-age strength but also more pronounced later-age enhancement.

When the NMR results are considered together with the TG and XRD findings, a coherent mechanistic picture emerges. XRD showed that GO refined the crystallite size of CH, while TG demonstrated that GO reduced the retained CH content, especially at higher fly ash dosage. The ^29^Si MAS NMR results further reveal where the consumed CH was likely utilized: it contributed to the development of a more polymerized silicate gel structure. In the fly ash-blended systems, CH acts as a key reactant in the pozzolanic reaction, and the conversion of CH into additional C-(A)-S-H gel is expected to increase both structural continuity and binding efficiency. Therefore, the higher MCL values and the increased Q^3^ + Q^4^ fractions in the GO-modified mixtures provide direct molecular-scale evidence that GO enhanced the later-age reaction of fly ash and promoted gel network maturation.

Overall, the ^29^Si MAS NMR analysis confirms that GO played a dual structural role in the fly ash-blended cementitious system. First, it reduced the fraction of less-reacted silicate species, indicating a more advanced reaction degree. Second, and more importantly, it promoted the formation of longer and more polymerized silicate chains, especially in the high-fly-ash system. These changes imply that the GO-modified mixtures developed a more mature C-(A)-S-H gel structure, which is expected to provide a stronger and more continuous load-bearing skeleton. This molecular-level evidence offers a critical link between hydration product evolution and the macroscopic compressive strength enhancement discussed earlier.

### 2.5. SEM-EDS Observations and Multiscale Mechanism Discussion

**Figure 7:**
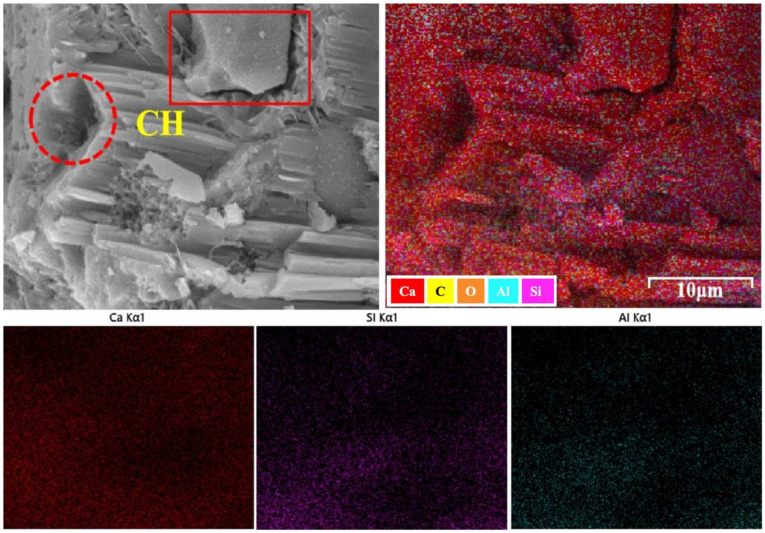
Micrograph and element mapping of G0F30.

**Figure 8:**
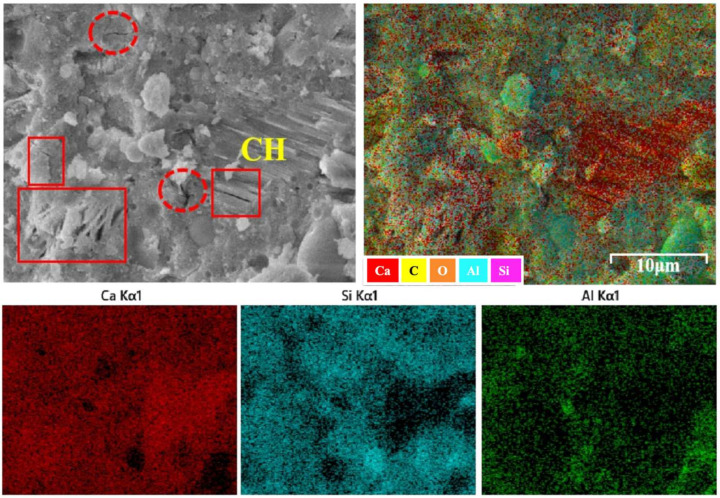
Micrograph and element mapping of G7F30.

**Figure 9:**
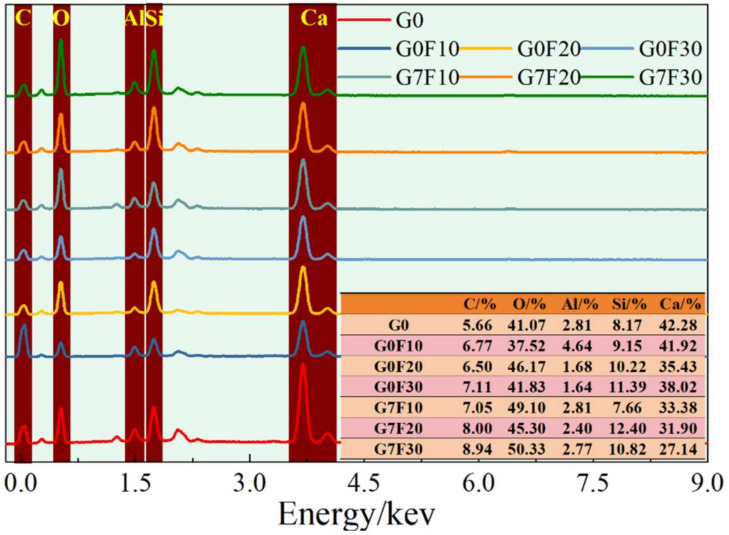
EDS elemental mapping results of fly ash-only samples and fly ash-GO co-doped samples.

**Figure 10:**
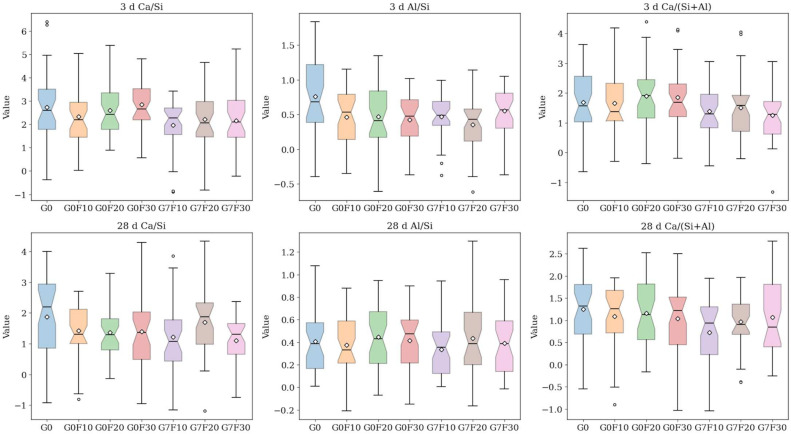
Boxplot comparison of EDS-derived elemental ratios for mixtures at 3 d and 28 d.

Figure 7, Figure 8 and Figure 9 shows the SEM morphologies and elemental mapping results at 28 days, where the left-hand images correspond to G0F30 and the right-hand images correspond to G7F30. In addition, the EDS results presented in Figure 10 provide the average values and standard deviations of Ca/Si, Al/Si, and Ca/(Si + Al) at 3 and 28 days for the fly ash-blended mixtures with and without GO. These results offer important morphological and compositional evidence for understanding the microstructural evolution induced by fly ash and GO.

At 28 days, the SEM image of G0F30 shows a relatively heterogeneous microstructure. Large plate-like CH crystals are still clearly visible, as highlighted in the left-hand micrograph, and some local pores or loosely packed regions can also be identified. The coexistence of coarse CH and discontinuous matrix regions indicates that, although hydration and pozzolanic reaction had progressed by 28 days, the microstructure of the GO-free high-fly-ash mixture was still not fully homogenized. The corresponding elemental mapping also shows that Ca is relatively concentrated in certain local domains, whereas Si and Al are less uniformly distributed. This non-uniform elemental distribution is consistent with the presence of CH-rich regions and a matrix in which the gel phase is not yet fully continuous.

By contrast, the G7F30 micrograph exhibits a markedly denser and more integrated matrix. Compared with G0F30, the amount of coarse plate-like CH appears reduced, while the surrounding matrix shows a more compact gel-like morphology with fewer obvious loose regions. Although some residual CH can still be identified, it is less dominant and more embedded within the surrounding hydration products. The elemental maps further show a more homogeneous distribution of Si and Al in the GO-containing sample, while the Ca-rich domains appear less sharply localized than in G0F30. This suggests that GO promoted a more uniform spatial organization of hydration products and reduced the tendency for CH to segregate into coarse isolated crystals.

The EDS quantitative results provide additional support for this interpretation. At 3 days, the average Ca/Si ratio decreased from 2.808 for G0 to 2.634, 2.507, and 2.454 for G0F10, G0F20, and G0F30, respectively, indicating that the incorporation of fly ash reduced the calcium dominance in the hydration products and increased the relative contribution of silicate-rich phases. After GO addition, the Ca/Si values decreased further to 1.993, 1.975, and 1.938 for G7F10, G7F20, and G7F30, respectively. A similar trend was observed for the Ca/(Si + Al) ratio, which decreased from 1.626 for G0 to 1.755, 1.659, and 1.724 for the GO-free fly ash mixtures at 3 days, and then to 1.370, 1.452, and 1.254 for G7F10, G7F20, and G7F30. Although the GO-free values did not show strict monotonicity, the overall reduction induced by GO is clear. These results indicate that GO promoted the formation of hydration products with lower calcium content and relatively higher silicate/aluminate incorporation, which is usually associated with the development of C-(A)-S-H gels rather than CH-dominated microstructures.

At 28 days, this trend became even more pronounced. The Ca/Si ratio of G0 decreased to 1.757, reflecting the general progress of hydration and the increasing contribution of gel products over time. For the GO-free fly ash mixtures, the Ca/Si values further declined slightly to 1.557, 1.549, and 1.543 for G0F10, G0F20, and G0F30, respectively. After GO addition, the values decreased again to 1.417, 1.397, and 1.382 for G7F10, G7F20, and G7F30. Likewise, the Ca/(Si + Al) ratio decreased from 1.253 for G0 to 1.151, 1.127, and 1.093 for G0F10, G0F20, and G0F30, and then to 1.014, 0.938, and 1.029 for G7F10, G7F20, and G7F30. These lower Ca/Si and Ca/(Si + Al) ratios indicate that the hydration products in the GO-modified systems became more silicate- and aluminosilicate-rich, which is consistent with more advanced pozzolanic reaction and greater incorporation of fly ash-derived Si and Al into the gel structure [40,41,42].

The Al/Si ratio provides further information on the compositional evolution of the binding gel. At 3 days, the Al/Si ratio decreased from 0.726 for G0 to 0.501, 0.511, and 0.423 for G0F10, G0F20, and G0F30, respectively, and to 0.454, 0.360, and 0.546 for G7F10, G7F20, and G7F30. At 28 days, the Al/Si values were 0.402 for G0, 0.352–0.412 for the GO-free fly ash mixtures, and 0.343–0.489 for the GO-containing mixtures. Although the Al/Si trend was not as regular as the Ca/Si trend, the combined EDS evidence still indicates that the GO-modified systems favored a gel chemistry with lower Ca enrichment and stronger participation of silicate/aluminosilicate units. Considering that fly ash provides both reactive silica and alumina, this compositional shift strongly supports the conclusion that GO facilitated the incorporation of fly ash-derived species into the hydration products.

Another important observation concerns the standard deviations of Ca/Si and Al/Si. In the 3-day GO-free mixtures, the Ca/Si standard deviation ranged from 0.956 to 1.101, whereas in the corresponding GO-containing mixtures, it ranged from 0.982 to 1.262. At 28 days, the Ca/Si standard deviation of G7F30 decreased to 0.843, lower than the value of 1.075 for G0F30. This reduction suggests that, at least in the high-fly-ash mixture, GO improved the later-age compositional uniformity of the matrix. This observation is in good agreement with the elemental mapping, where G7F30 exhibits a more homogeneous distribution of Si and Al and less obvious segregation of Ca-rich CH domains. Therefore, GO appears not only to modify the average gel chemistry, but also to improve the spatial uniformity of the hydration products in the matrix.

When the SEM–EDS results are integrated with the XRD, TG, and ^29^Si MAS NMR analyses, the strengthening mechanism of GO becomes much clearer. XRD demonstrated that GO reduced the crystallite size of CH, while TG showed that the retained CH content decreased, especially in the high-fly-ash mixture. The ^29^Si MAS NMR results further revealed increased MCL and enhanced Q^3^ + Q^4^ fractions in the GO-modified systems, indicating a more polymerized silicate gel structure. The SEM–EDS observations presented here provide direct microstructural confirmation of these changes: the GO-containing matrix is denser, coarse CH is less dominant, and the elemental distribution is more compatible with the formation of silicate/aluminosilicate-rich gels. Therefore, the role of GO can be summarized as a coupled regulation effect involving CH refinement, CH conversion, gel polymerization, and matrix homogenization.

For the high-fly-ash system represented by G7F30, this coupled effect is particularly significant. In the GO-free G0F30 sample, the microstructure still contains relatively coarse CH and locally heterogeneous regions at 28 days, indicating incomplete coordination between cement hydration and fly ash reaction. In contrast, the GO-modified G7F30 sample shows a denser matrix, lower Ca/Si ratio, lower Ca/(Si + Al) ratio, and higher MCL, all of which point to a more mature C-(A)-S-H gel network. This microstructural evolution explains why G7F30 achieved the highest 28-day compressive strength among all the investigated mixtures. Thus, the SEM–EDS results provide the final morphological and compositional evidence that GO effectively enhanced the utilization of fly ash and promoted the formation of a stronger and more continuous binding framework. Accordingly, the enhancement in compressive strength by GO should be interpreted as the consequence of multiscale structural regulation, including CH refinement, promoted CH utilization, silicate gel polymerization, and matrix densification, rather than a simple filler or dispersion effect.

## 3. Conclusions

This study systematically investigated the age-dependent role of GO in fly ash-blended cementitious materials through compressive strength, XRD, TG-DTG, ^29^Si MAS NMR, and SEM-EDS analyses. Based on the obtained results, the following conclusions can be drawn:

1. The incorporation of fly ash reduced the early-age compressive strength of the cementitious system, mainly due to the clinker dilution effect and the relatively low early pozzolanic reactivity of fly ash. However, the strength difference between the fly ash-blended mixtures and the plain cement reference became much smaller at 28 days, indicating that the later pozzolanic contribution of fly ash gradually compensated for the early-age strength loss.
2. The addition of 0.07% GO significantly enhanced the compressive strength of fly ash-blended cementitious materials at all investigated ages. At 3 days, GO effectively compensated for the adverse influence of fly ash on early strength development, and at 28 days, the GO-modified mixtures exhibited much higher compressive strengths than their GO-free counterparts. The greatest enhancement was observed in the high-fly-ash system, demonstrating that GO was particularly effective in improving the structural development of mixtures with relatively high fly ash content.
3. XRD and TG-DTG results consistently showed that both fly ash incorporation and GO addition altered the evolution of Ca(OH)_2_. Fly ash reduced the retained CH content because of both cement dilution and pozzolanic consumption, while GO further refined the CH crystallization state and decreased the residual CH content, especially in the high-fly-ash mixture. These results indicate that GO promoted a more efficient utilization and conversion of CH during hydration and subsequent fly ash reaction.
4. ^29^Si MAS NMR analysis revealed that GO promoted the development of a more mature and polymerized silicate gel structure. Compared with the GO-free mixtures, the GO-modified systems exhibited lower Q0 fractions, higher MCL, and, in particular, an increased proportion of Q3 + Q4 species in the high-fly-ash mixture. This demonstrates that GO not only accelerated the formation of hydration products, but also enhanced the later-age polymerization and structural evolution of C-(A)-S-H type gels.
5. SEM-EDS observations further confirmed that GO improved the microstructural integrity and compositional characteristics of the fly ash-blended matrix. At 28 days, the GO-modified high-fly-ash sample exhibited a denser morphology, less dominant coarse CH, and lower Ca/Si as well as Ca/(Si + Al) ratios, indicating a stronger incorporation of fly ash-derived Si and Al into the hydration products. These features are consistent with the formation of a more continuous and stable binding framework.

Overall, the strengthening effect of GO in fly ash-blended cementitious materials should be understood as a multiscale regulation process rather than a simple physical filling effect. GO simultaneously promoted early hydration product nucleation, refined and utilized CH more effectively, enhanced silicate gel polymerization, and improved matrix densification. Through these coupled effects, GO effectively compensated for the early-age weakness of fly ash systems and significantly improved their later-age mechanical performance. Further work should focus on GO dosage optimization, fresh-state rheology and dispersion behavior, and long-term performance at later curing ages in order to extend the applicability of the present mechanism-based observations.

## 4. Materials and Method

### 4.1. Materials

**Figure 11:**
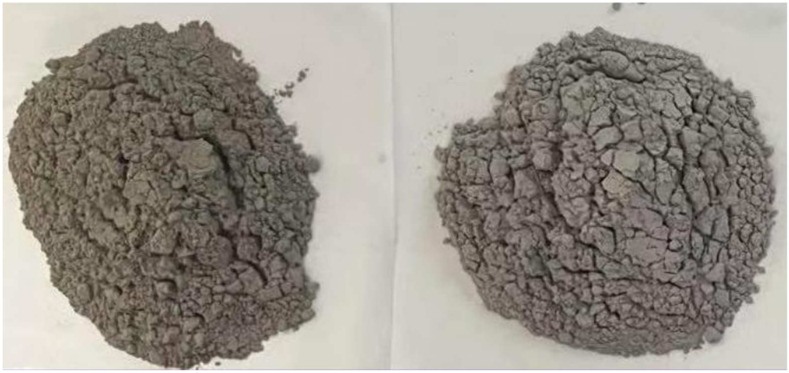
Images of raw materials: cement (**left**), fly ash (**right**).

Ordinary Portland cement (OPC, P.O 42.5) was used as the primary binder in this study. Fly ash was used as the supplementary cementitious material to partially replace cement at different replacement levels. Figure 11 presents the images of cement and fly ash. GO was employed as the nano-modifying additive. The GO was supplied by Suzhou Tanfeng Graphene Technology Co., Ltd. (Suzhou, China), with a lateral size of about 10–50 μm, a thickness of 1–2 layers, a BET specific surface area of 132.55 m^2^/g, and a purity higher than 95 wt.%. ISO standard sand was used for strength specimens [43], while deionized water was used for sample preparation. A polycarboxylate ether superplasticizer (PCE) was used to assist GO dispersion and to maintain comparable workability among mixtures. Specifically, GO tends to agglomerate in cement systems because the high-pH pore solution compresses the electrical double layer, while dissolved Ca^2+^ can interact with oxygen-containing functional groups on adjacent GO sheets and promote inter-sheet bridging and flocculation. Previous studies have identified high alkalinity and calcium ions as the main causes of GO agglomeration in cementitious environments [44,45]. PCE alleviates this problem mainly through adsorption-assisted steric stabilization. Reported studies show that PCE molecules can adsorb onto GO surfaces in cement pore solution, and their side chains provide steric hindrance that reduces direct sheet-to-sheet restacking. At the same time, the interaction among GO, Ca^2+^, and PCE forms a more stable interfacial adsorption structure, which improves the dispersion stability of GO in the ionic cement environment. Experimental evidence has further shown that PCE can effectively remedy the agglomeration behavior of GO in cement pore solution, and that higher charge density and longer side chains are more favorable for dispersion [46,47,48]. The selection of fly ash was motivated by its ability to reduce clinker consumption and associated carbon emissions, although its lower early reactivity is known to delay hydration and reduce early-age strength. GO was introduced because of its high specific surface area and abundant oxygen-containing functional groups, which can provide heterogeneous nucleation sites for hydration products and regulate the development of silicate gel structures. In the present work, the fly ash dosage was varied at 10%, 20%, and 30% by mass of binder, and the GO dosage was fixed at 0.07 wt.% of total binder. The detailed chemical compositions of cement and fly ash determined by X-ray fluorescence analysis are reported in Table 1.

### 4.2. Sample Preparation

**Figure 12:**
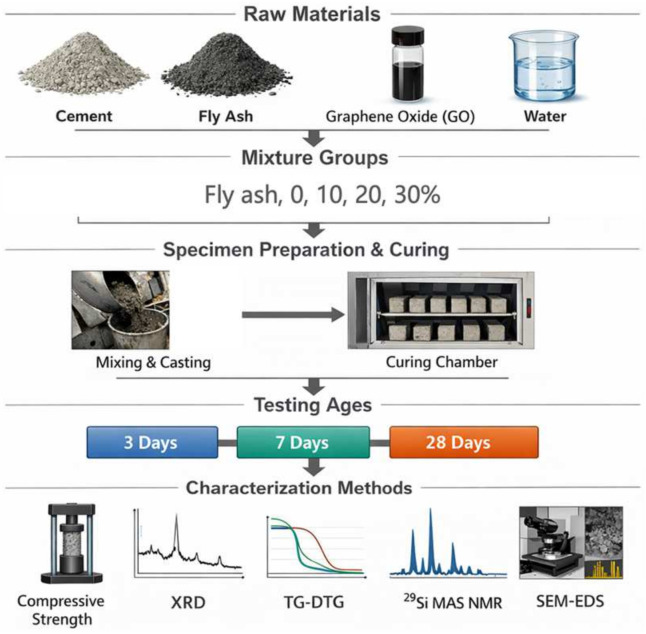
Schematic diagram of the experimental program.

To investigate the combined effects of fly ash dosage and GO incorporation, one control mixture without fly ash or GO was first prepared and denoted as G0. Fly ash was then introduced at replacement levels of 10%, 20%, and 30% by mass of binder, and the corresponding mixtures without GO were denoted as G0F10, G0F20, and G0F30, respectively (see Table 2). For the GO-modified mixtures, 0.07 wt.% GO by mass of total binder was incorporated into the fly ash-blended systems, and the corresponding mixtures were denoted as G7F10, G7F20, and G7F30, respectively. For mechanical testing, mortar specimens were prepared according to the general framework. The total binder content was fixed at 450 g, the mass of ISO standard sand was 1350 g, and the water-to-binder ratio was fixed at 0.4. The dosage of PCE was adjusted slightly, within 0.10–0.20 wt.% of binder, only to ensure a consistent flow condition after GO incorporation. The schematic diagram of the experimental program is illustrated by Figure 12.

To minimize GO agglomeration and ensure effective nanosheet dispersion, the required amount of GO was first dispersed in part of the mixing water containing the predetermined amount of PCE. The suspension was then ultrasonicated for 30 min using an ultrasonic disperser operating at approximately 300 W. This procedure was used to promote exfoliation and homogeneous distribution of GO in the aqueous medium before contact with the binder, as suggested by previous studies [12,13,49]. Cement and fly ash were dry-mixed for 2 min to ensure uniform blending. The GO-containing solution, or plain mixing water for GO-free mixtures, was then gradually introduced into the dry blend and mixed in a mortar mixer. The mixing procedure consisted of low-speed mixing for 30 s, a rest period of 30 s for scraping the bowl wall, and high-speed mixing for 60 s. Afterward, standard sand was added gradually, and mixing continued for another 60 s at low speed and 60 s at high speed until a homogeneous mortar was obtained. The fresh mortar was cast into 40 × 40 × 40 mm^3^ cubic molds for strength testing. After casting, the molds were vibrated to remove entrapped air and covered with plastic film to prevent moisture loss. All specimens were demolded after 24 h and then cured in a standard curing chamber at 20 ± 2 °C and relative humidity ≥95% until the designated ages of 3, 7, and 28 d. At the target age, small interior fragments were taken from the paste specimens. Hydration was stopped by immersion in anhydrous ethanol for 24 h, after which the ethanol was refreshed and the samples were stored for an additional 6 d to ensure adequate solvent exchange. The samples were then dried in a vacuum oven at 60 °C for 24 h to remove residual solvent and physically adsorbed moisture while minimizing microstructural damage. After drying, the samples were treated differently depending on the characterization technique. For XRD and TG-DTG, the fragments were ground to a fine powder and passed through a 45 um sieve. For ^29^Si MAS NMR, the dried sample was finely crushed and packed into zirconia rotors. For SEM-EDS, representative fragments from the interior region were selected, mounted, and coated with a gold before testing.

### 4.3. Testing

The compressive strength was determined at curing ages of 3, 7, and 28 d using the prism mortar specimens described above. The test was conducted according to GB/T 17671-2021 [50]. Each cubic specimen was subjected to compressive testing with the loading rate controlled at 2.4 kN/s. For each mixture and test age, at least three specimens were prepared and the mean value of compressive strengths was reported. The compressive strength data were used to assess the combined effects of fly ash dosage and GO incorporation on the development of the mechanical performance of the investigated cementitious materials.

X-ray diffraction analysis was performed to identify the main crystalline hydration products and to evaluate the crystallization behavior of Ca(OH)_2_ (CH). Powdered samples were tested using an X-ray diffractometer with Cu K-alpha radiation (lambda = 1.5406 A), operated at 40 kV and 40 mA. The diffraction patterns were collected over a 2 theta range of 5–70 degrees with a step size of 0.02 degrees and a scanning rate of 4 degrees/min. The main crystalline phases were identified by comparison with standard PDF cards. Particular attention was paid to the characteristic CH peaks. The average CH crystallite size was estimated using the Scherrer equation, based on selected CH diffraction peaks. This parameter was used to compare the refinement effect of fly ash and GO on the CH phase.

Thermogravimetric analysis (TG-DTG) was carried out to quantify the decomposition behavior of hydration products and to estimate the retained CH content in the hardened paste. Approximately 10 mg of powder was placed in an alumina crucible and heated from ambient temperature to 1000 °C at a heating rate of 10 °C/min under a nitrogen atmosphere flowing at 50 mL/min. The mass losses associated with dehydration of bound water, decomposition of CH, and decomposition of CaCO_3_ were identified from the TG-DTG curves. The total CH content (M_total) was calculated from the mass loss in the CH decomposition region using the stoichiometric relationship between Ca(OH)_2_ and H_2_O. In addition, the theoretical CH generated by the cement fraction (M_ce) and the CH variation associated with the mineral admixture (M_MA) were calculated to evaluate the interaction between cement hydration and fly ash reaction.

^29^Si magic-angle spinning nuclear magnetic resonance (^29^Si MAS NMR) was used to characterize the local silicate environments and the structural evolution of hydration gels. The spectra were recorded on a solid-state NMR spectrometer at a ^29^Si resonance frequency of 79.49 MHz using a 7 mm zirconia rotor. The spinning speed was set to 5 kHz, the recycle delay was 60 s, and 2000–4000 scans were accumulated to obtain an adequate signal-to-noise ratio. Tetramethylsilane was used as the chemical-shift reference. The spectra were deconvoluted into Q^0^, Q^1^, Q^2^, Q^3^, and Q^4^ components. Here, Q^0^ represents isolated silicate tetrahedra typically associated with less-reacted silicate units, Q^1^ and Q^2^ correspond to the end-chain and middle-chain tetrahedra in C-(A)-S-H type gels, and Q^3^-Q^4^ indicate more polymerized silicate structures. Based on the deconvolution results, the relative fractions of different Q^n^ species were obtained, and the mean chain length (MCL) was calculated to evaluate the degree of silicate polymerization.

Microstructural morphology and elemental distribution were examined by field-emission scanning electron microscopy coupled with energy-dispersive spectroscopy (SEM-EDS). Dried interior fragments were mounted on aluminum stubs and sputter-coated with gold. SEM observations were carried out at an accelerating voltage of 15 kV and a working distance of approximately 10 mm. For elemental mapping and compositional analysis, EDS was used to determine the spatial distributions of Ca, Si, Al, and O. In addition, 30 random point analyses were collected for each selected specimen to calculate the atomic ratios of Ca/Si, Al/Si, and Ca/(Si + Al). The corresponding mean values and standard deviations were used to assess the average gel chemistry and compositional uniformity of the hydration products.

## Figures and Tables

**Table 1:** Chemical compositions of cement and fly ash.

Oxide	Cement	Fly Ash	Unit
SiO_2_	20.8	53.5	wt.%
Al_2_O_3_	5.3	26.2	wt.%
CaO	63.8	5.2	wt.%
Fe_2_O_3_	3.4	7.8	wt.%
MgO	2.1	1.4	wt.%
SO_3_	2.7	0.5	wt.%
Na_2_O	0.2	0.4	wt.%
K_2_O	0.6	1.8	wt.%
TiO_2_	0.3	1.1	wt.%
P_2_O_5_	0.1	0.3	wt.%
MnO	0.1	0.1	wt.%
Loss on ignition (LOI)	0.6	1.7	wt.%
Total	100	100	wt.%

**Table 2:** Mix proportions of fly ash-only and fly ash- GO composite samples (g).

Notation	Water	Cement	Fly Ash	Sand	GO	PCE
G0	180	450	0	1350	0	0.45
G0F10	180	405	45	1350	0	0.45
G0F20	180	360	90	1350	0	0.45
G0F30	180	315	135	1350	0	0.45
G7F10	180	405	45	1350	0.32	0.68
G7F20	180	360	90	1350	0.32	0.68
G7F30	180	315	135	1350	0.32	0.68

## Data Availability

Data will be available on request.
